# How good is the Myers-Briggs Type Indicator for predicting leadership-related behaviors?

**DOI:** 10.3389/fpsyg.2023.940961

**Published:** 2023-03-02

**Authors:** Rodrigo Zárate-Torres, Juan C. Correa

**Affiliations:** ^1^Colegio de Estudios Superiores de Administración, Bogotá, Colombia; ^2^Tecnológico de Monterrey, Monterrey, Mexico

**Keywords:** leadership, leadership practices inventory, LPI, MBTI, personality, reproducible research

## Abstract

The Myers-Briggs Type Indicator (MBTI) is a popular tool used by psychologists working as managers' coaches in organizational contexts. Despite its popularity, few studies provide empirical evidence on the role of the MBTI as a predictor of managers' leadership-related behaviors. This article is written based on research that answers the question of how good the MBTI is to prove leadership behavior. It does so by comparing goodness-of-fit indexes of two confirmatory factor analysis models and two structural models on the personality-leadership relationship, following standards of reproducible research principles. We sampled 529 participants who were graduate and undergraduate students enrolled in business administration programs from Colombian universities. Results show conclusive evidence of the psychometric measurement of both MBTI and leadership practices, even though the relationship between MBTI and the leadership practices inventory proved to be weak.

## 1. Introduction

The Myers-Briggs Type Indicator (MBTI) is well-known in psychology and related fields as a self-report questionnaire. Its development relied on Jung's seminal ideas on psychological types as a framework to describe human personality (Jung, 1923). Nowadays, MBTI is a tool that provides a variety of practical purposes. Credit scores prediction (Ertemel and Çaylak, 2021), analysis of construction workers' safety behavior (Ma et al., 2021), validation of artificial intelligence techniques (Sahono et al., 2020; Genina et al., 2021), or prediction of judging-perceiving behaviors in online social forum (Choong and Varathan, 2021) are just a few recent examples of such purposes.

In business and managerial contexts, the reputation of MBTI probably emerged in the last decade of the last century (Schweiger, 1985; Furnham and Stringfield, 1993; Zumbo and Taylor, 1993; Ramsoomair, 1994; Mani, 1995) when its utility as a professional tool was summarized by Gardner and Martinko (1996). Among the many usages of MBTI, one of the most relevant is its value as a data collection tool to understand leaders' differences, let alone its value in promoting self-awareness of their behaviors and boosting team learning and development in organizational settings (Costello, 1993; Pestana and Codina, 2019, 2020; Penzias, 2020). Even though the MBTI is useful for teaching leadership skills (Shope et al., 2000), we are not aware of previous endeavors focusing on assessing its strengths as a leadership predictor. To the best of our knowledge, previous works have focused on literature reviews (Gardner and Martinko, 1996; Brown and Reilly, 2009) while the study of Bess and Harvey (2002) emerged as one of the few statistical studies that analyzed the bimodal empirical distributions that result from using this tool as a data collection technique. The aim of the current article is to analyze the strength of the relationship between personality, as described by the MBTI, and leadership performance. The research was conducted by resuming the conceptual ideas that pinpoint the link between leadership-related behaviors with the MBTI scores that describe personality dichotomies. To do this, we sampled 529 participants who were graduate and undergraduate students enrolled in business administration programs from Colombian universities. Apart from continuing this research orientation, the contribution of this article is visible in terms of principles of open science, as it incorporates reproducible research standards that facilitate the audit of findings and maximizes the chances of reproducibility in further studies (Hardwicke et al., 2022).

### 1.1. Personality dichotomies-MBTI

The use of MBTI as a tool for identifying personality characteristics in university students is not novel and the work of Zavyalova et al. (2021) with Russian students illustrates it. Nonetheless, we are not aware of similar efforts in Latin-American countries. As per Choong and Varathan (2021) there are two approaches to personality, the trait-based approach and the type-based approach. The so-called *Big Five*, created by Srivastava et al. (2003), is probably the best-known measurement instrument that relied on the trait-based approach that posed the idea of five large dimensions of personality, hence its name in its original English language version. In contrast, the MBTI is the best-known measurement tool that relied on the type-based approach.

The MBTI is a forced-choice instrument designed to determine how people see the world and how people make decisions (Myers and Mccaulley, 1985; Myers, 1993, 2016, 2019; King and Mason, 2020). It was created by Katherine and Isabel Briggs following Carl Jung's personality theory (Myers and Mccaulley, 1985; Myers, 1993; King and Mason, 2020). The MBTI consists of four opposite dichotomies (Myers and Myers, 2010), *Extraversion-Introversion*, that refers to where to focus attention and energy, introverts focus their energy inside of them and they are interested in the world of thoughts and reflections while extroverts focus their attention and energy outward, and they are interested in the world of people and things; *Intuition-Sensing*, that refers to what kind of information people like and trust, sensitives prefer to take information using their five senses while intuitive people go beyond what is real or concrete and focus on meaning, associations, and relationships; *Feeling-Thinking*, that refers to the way people make decisions, feelers make their decisions with a person-centered, values-based process while thinkers make their decisions based on impersonal, objective logic, and finally *Judgment-Perceiving*, that relates to the way we orient ourselves to the external world, people who prefer judgment want the external world to be organized and orderly while people who prefer perceiving, seek to experience the world, not organize it (Choong and Varathan, 2021). When combining the four dichotomies, extroversion-introversion, intuition-sensing, feeling-thinking, judgment-perceiving, it results in sixteen types of personalities (i.e., ISTJ, ISTP, ISFJ, ISFP, INTJ, INTP, INFJ, INFP, ESTJ, ESTP, ESFJ, ESFP, ENTJ, ENTP, ENFJ, and ENFP) (King and Mason, 2020). The MBTI has received criticism in basically one way from trait theorists: people can not be classified dichotomously (King and Mason, 2020). Despite of this criticism, MBTI researchers have shown different studies with acceptable indexes of reliability and validity (Furnham and Stringfield, 1993; King and Mason, 2020) and this has paved the way to its continuing use in research across the world as shown by Garland and Village (2021).

### 1.2. Leadership practices inventory

Academics have traditionally studied leadership from two perspectives, one that focuses on positional leadership within an organization's hierarchy and one that views leadership as a process of social influence that occurs naturally in a social system (Helland and Winston, 2005). Within these perspectives, four approaches to leadership theories have been determined: traits, behaviorist, contingency, and transformational (Helland and Winston, 2005). According to Helland and Winston (2005), all of these approaches have examined the traits and behaviors of leaders, how they employ power and influence, and how they adapt their behavior to particular situations. According to Van Maurik (2001), none of these four approaches is mutually exclusive or is determined for a certain period of time only.

Bass and Riggio (2006) refer to the Leadership Practices Inventory (LPI) as a measurement instrument for transformational leadership taught in leadership development programs.

The seminal conception, empirical development, and psychometric validation of the LPI were the resulting work of Posner and Kouzes (1988) who based this approach from Burns (1978). The original design of LPI relied on systematic feedback and interviews from participants that were surveyed with a set of behaviorally-based statements under Burn's ideas of *Transformational leadership* as contrasted with *Transactional leadership*. As per Burns (1978), transformational leadership is a process in which leaders and followers help each other to reach a higher level of morale and motivation, whereas transactional leadership has more to do with the use of rewards and punishments to shape and promote followers' compliance. Existent meta-analyzes focusing on comparing both leadership styles have shown that transformational leadership seems to have stronger correlation with teams' productivity (Lowe et al., 1996), commitment, role clarity, and well-being as compared with transactional leadership (Tafvelin, 2013). More recent studies revealed that transformational leadership and the behavior of sharing knowledge proved to be uncorrelated from supervisors' perspective, whereas they are correlated from collaborators' perspective (Durán and Castañeda, 2015).

The LPI evaluates five leadership behaviors that are considered exemplary behaviors of leaders (Zárate-Torres and Matviuk, 2012; Kouzes and Posner, 2019). These behaviors are: 1) Challenging the process: This practice refers to questioning the status quo, seeking innovation, finding opportunities, taking risks, and learning from experience. 2) Inspire a shared vision: This practice refers to how often the leader shares or describes the vision to his followers if he involves his followers informing that vision and the passion with which he shares that vision. 3) Enabling others to act: This practice refers to empowering followers, fostering collaboration, and delegation. 4) Modeling the way: This practice refers to the example that the leader gives and the clarity in his values and his knowledge of himself and the consistency between what he says and what he does and how he lives and manifests his values. 5) Encouraging the heart: This last practice refers to the recognition made by the leader both public and private of the individual and group achievements.

### 1.3. The impact of personality on leadership

The personality-leadership relationship is perhaps one of the most relevant relationships in the contemporary literature of management (Luong et al., 2021; Spark and O'Connor, 2021; Harvey and Green, 2022; Perret and Powers, 2022). According to Bass et al. (1990), some definitions conceptualize leadership from a personality perspective that suggests that leadership is a combination of special traits or characteristics possessed by an individual, and they empower a person to influence others to achieve a goal. Nonetheless, in the work of Bass et al. (1990), there is no single piece of empirical evidence that shows the direct impact that personality has on influencing others to achieve a common goal. As per Waite and McKinney (2015), when one person recognizes his/her personality preferences using a self-assessment tool, he or she has a critical input that facilitates his/her leadership development. Thus, it becomes highly relevant to quantify the link between personality and leadership, for the following reasons.

According to Arévalo-Avecillas et al. (2019) the United States of America has produced most of the empirical studies tackling the relationship between personality and leadership, and although Norway, Australia, Singapore, and Canada have shown a few other set of studies on the correlation between these two constructs, no similar efforts are evident from developing countries (Arévalo-Avecillas et al., 2019). In a recent study, Zárate-Torres et al. (2022) reported the scarcity of Latin-American studies focusing on the relationship between personality, gender, and leadership. Likewise, Chacón-Henao et al. (2022) reported that in emerging Latin-American countries such as Colombia, leadership studies have focused mainly on particular attributes and styles of individual leaders (Hincapié-Montoya et al., 2018; Reyes Bastidas and Briano-Turrent, 2018; Gaviria-Rivera and López Zapata, 2019; Rojero-Jiménez et al., 2019). We regard these recent studies as evidence that shows how leadership studies in Colombia and Latin America countries deserve additional local efforts to unveil how leadership and personality proved to be linked when using tools such as the MBTI and the LPI.

When partitioning leadership into their behavioral practices and personality into their dichotomies, one can identify some patterns of interest. For example, Spark and O'Connor (2021) has provided evidence about the relationship between state extraversion and emergent leadership, as extraverts are more likely than introverts to emerge as leaders (Colbert et al., 2012). In practical terms, this means that introverts face more difficulties in growing professionally and reaching leadership roles. This argument is also probed by the study of Luong et al. (2021) where they compared extraverts and introverts and the challenges they faced, suggesting that personality matters when leading. Another token that illustrates this point, is the study of Garland and Village (2021) who used MBTI to classify people in leadership positions and found that intuitive leaders are more visionaries that sensitive leaders (e.g., sensitives have clear guidelines on expectations roles and responsibilities, while intuitives find opportunities to participate in designing the future Myers and Mccaulley (1985)). Likewise, Zhang et al. (2021) suggested that in order to enhance team effectiveness, managers should balance team members in terms of judgers and perceivers. For example, when all members are perceivers, they will face problems with deadlines definitions; in comparison, when all members are judgers they will find difficulties how to handle unexpected contingencies. Until this point, it should be evident that the personality-leadership link deserves scrutiny in its own right. A note of consideration is worth mentioning here. In real-world scenarios, a manager is more concerned with what works than with ultimate theoretical developments. Although such a vision is certainly distant from an orthodox academic perspective, its soundness in team performance is of highly relevance. For example, when a manager detects that his/her subordinates are more sensing (e.g., they follow the norms and procedures) than intuitive (i.e., they look for alternative ways to introduce innovations in the organization, regardless these innovations exist in current norms and procedures), the ideal leader understands how to work as a team with his/her followers.

To evidence the above, in the literature, several studies show that MBTI dichotomies relate to leaders' behaviors in general (Connor et al., 2014; Uusi-Kakkuri and Brandt, 2015), transformational leaders' behavior in particular (Brown and Reilly, 2009), and leadership practices as captured by LPI (Zárate-Torres et al., 2022). All these behaviors have important implications for effective leadership. In their MBTI manual (chapter 13) Myers and Mccaulley (1985), pinpoint several practical uses of this tool, such as improving communications in firms, conflict resolutions, enhancement of problem-solving and decision-making, planning, implementing, and managing organizational change, and improving teams' productivity.

## 2. Materials and methods

Our methodological approach is similar to previous efforts that explored the relationship between personality and leadership qualities (Zhang et al., 2021), although it focuses on a different framework to address other goals. In the current article, we sampled 529 participants who were graduate and undergraduate students enrolled in business administration programs from Colombian universities. After data cleaning and preprocessing, we obtained a total of 464 valid observations of which 50.64 % were from men, and 48.06 % from women. 7.11 % of the participants were students in their last semester of their undergraduate program, while 91.6 % were graduate students. 15.94 % of the participants were between 15 and 21 years old, 47.41% aged between 26 and 35 years old, 24.56% were between 36 and 45 years old, and 10.77% were older than 46. Data collection relied on valid and already translated questionnaires in their Spanish version. Also, participants took the instruments at different times and days during class hours. Questionnaire administration was conducted by the principal investigator, who was always present in the classroom to answer any questions from participants and prevent confounding factors that could emerge during questionnaire administration and lead to problems of uncontrolled statistical variance (Rodríguez-Ardura and Meseguer-Artola, 2020).

As compared with previous endeavors that explored leadership practices in relation to emotional intelligence from Colombian samples (Zárate-Torres and Matviuk, 2012), we are unaware of specific studies tackling leadership practices in relation to personality dichotomies in Colombia. The focus on Colombia is highly relevant as compared with samples from other countries, because Colombia joined the organiczation for Economic Co-operation and Development, OECD, in April 28, 2020 (Sakiru et al., 2022). Indeed, the so-called soft skills (e.g., leadership, teamwork, creativeness) are of paramount importance, as suggested by previous OECD reports (OECD, 2016) because these skills allow the future generation of Colombian businessmen to compete in a more globalized context where Colombia is expected to leapfrog its current dynamic capacities. As individuals need to be trained in how to compete, higher education executive programs are the standard societal mechanism that paves the way for the next generation of managers to succeed (Núñez et al., 2012). In our view, teaching leadership as a soft skill provides an incomplete landscape for students if the leadership-personality link is not clearly defined.

### 2.1. Data collection procedure

All participants were told about the purpose of this research and provided their consent agreement to participate in the study. We documented supplemental material following reproducible research standards to increase data transparency and results reproducibility (Peikert and Brandmaier, 2021; Peer et al., 2022). We used MBTI and the Leadership Practices Inventory (LPI) as data collection techniques. The MBTI consists of 93 items and normally takes between 20 and 40 min to complete (https://www.humanmetrics.com/personality/test). The score of the questionnaire provides participants with their 4-letter personality type. The first type (P1) is *Extraversion-Introversion*, the second type (P2) is *Intuition-Sensing*, the third type (P3) is *Feeling-Thinking*, and the fourth type (P4) is *Judgment-Perceiving*. The LPI consist of 30 questions (https://cpb-us-w2.wpmucdn.com/u.osu.edu/dist/5/60574/files/2018/05/LPI-plus-scoring-guide-203il6w.pdf). These questions are distributed in the following five conceptual dimensions. The first dimension (L1) refers to *challenging the process*. The second dimension (L2) is *inspiring a shared vision*. The third dimension (L3) is *enabling others to act*. The fourth dimension (L4) is *modeling the way*, and the fifth dimension (L5) is *encouraging the heart*. Participants' responses were then tabulated in a standard data set that we used as computational input for statistical analyzes.

### 2.2. Statistical analyzes

The scrutiny followed in this article relies on the combination of bivariate correlation analyzes with the help of the R package psych (Revelle, 2022) and conventional confirmatory factor analyzes proposed by Bollen (1989) and implemented with the help of the lavaan R package (Rosseel, 2012). A note of consideration is worth mentioning here. It is widely accepted that for psychometric validation purposes, a standard approach is to rely on an exploratory factor analysis that reveals that the empirical structure is consistent with the theoretical structure and then apply a confirmatory factor analysis as an ultimate empirical test. This standard approach is only valid when the psychometric structure of the questionnaires or tests is unknown. When the psychometric structure of these tests is already known in advance, there is no need to rely on a preliminary exploratory factor analysis. In Bollen's terms “*in confirmatory factor analysis a model is constructed in advance, the number of latent variables is set by the analyst*” (p. 228).

We used the software Ωnyx (von Oertzen et al., 2015) to specify and identify structural equation models on the personality-leadership relationship. The syntaxes produced by Ωnyx were then exported and statistically tested in RStudio. We also used the R package semTable (Johnson and Kite, 2020) to facilitate the reporting of findings *via* LAT_E_Xreproducible documentation (Gandrud, 2018).

## 3. Results

**Figure 1:**
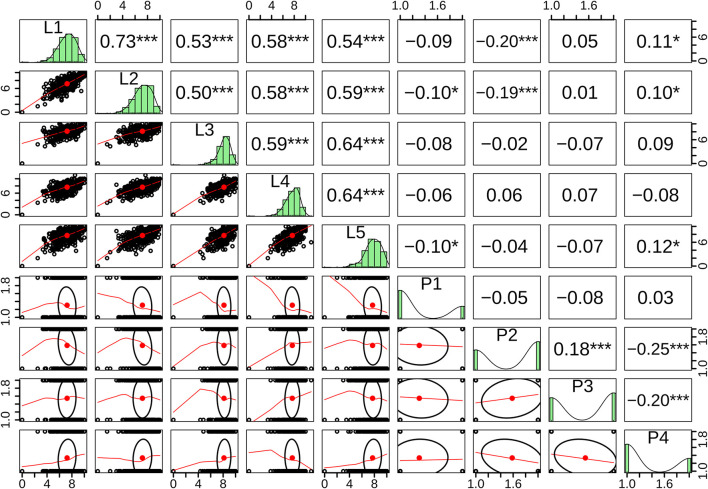
Spearman correlation matrix plot for the five items of leadership and four items of personality. ^*^the correlation is significant at 0.1; ^**^the correlation is significant at 0.05; ^***^the correlation is significant at or below 0.001.

We initialize the analysis by describing the univariate distribution of every single conceptual dimension for both the personality self-report of the Myers-Briggs Type Indicator (i.e., P1, P2, P3, and P4) and the Leadership Practices Inventory and its five dimensions (i.e., L1, L2, L3, L4, and L5). We observed that dichotomies assessing personality follow a Bernoulli distribution, while the practices assessing leadership follow a Binomial distribution. As these distributions are discrete and their parametric properties deviate from those of a Gaussian distribution, we evaluated their bivariate statistical behavior through the Spearman Correlation Matrix Plot as depicted in Figure 1. As expected, all correlations among leadership practices proved to be statistically significant (0.50 ≥ ρ ≤ 0.73, *p* < 0.01). Although correlations between dichotomies assessing personality were also statistically significant, their magnitude proved to be lower (−0.2 ≥ ρ ≤ 0.18, *p* < 0.01) and one item (i.e., P1) was not correlated with the rest.

The correlations above reveal interesting findings. Leadership practices share intermediate to high correlations among them. Nonetheless, MBTI dichotomies show low correlations among them. Above and beyond these patterns, it is worth mentioning how the leadership practices of *challenging the process* and *inspiring a shared vision* correlate with the MBTI dichotomy *Intuition-Sensation*. When people score high in these two leadership practices, they tend to fall as intuitive persons. In other words, intuitive persons tend to be more creative and this creativity allows them to challenge the processes around them. The *Extraversion-Introversion* dichotomy shows a low correlation with *inspiring a shared vision* and *encouraging the heart*. Particularly, leaders with higher scores on these two practices tend to be extroverted. We also noticed that the dichotomy *Judgement-Perception* shows a low positive correlation with two leadership practices: *challenge the process* and *inspiring a shared vision*, and a low negative correlation with *encouraging the heart*. This particular pattern of associations suggests that leaders with higher scores in the first two leadership practice tend to be perceivers while leaders with higher scores in *encouraging the heart* tend to be of judgement type. In order to test our main hypothesis that personality is a statistically significant predictor of leadership, we now proceed with reporting resulting confirmatory factor analyzes.

### 3.1. Confirmatory factor analyzes

**Figure 2:**
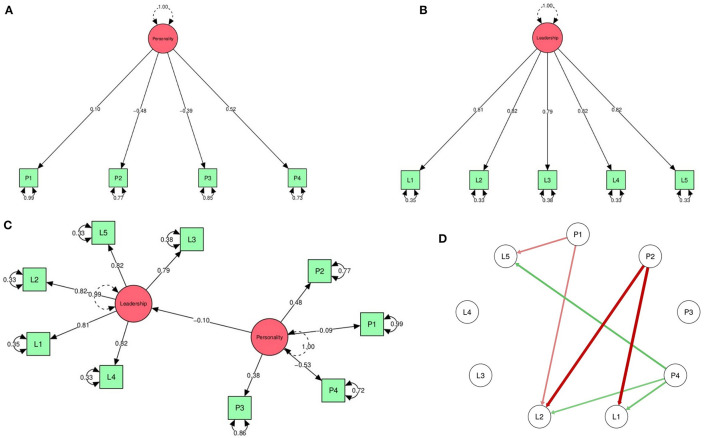
Models of **(A)** confirmatory Factor analysis for personality, **(B)** confirmatory factor analysis for leadership, **(C)** structural personality-leadership model, and **(D)** network visualization.

Our first confirmatory factor analyzes (CFA) relate to the personality self-report questionnaire (see Figure 2A). In Table 1 we present two statistical parameter estimations. In model 1, parameters were estimated through the Full Information Maximum Likelihood (FIML) estimation method, which can be regarded as a more restricted approach as it assumes that the observed indicators follow a continuous and multivariate normal distribution. In model 2, parameters were estimated through Diagonally Weighted Least Squares method (DWLS), which can be regarded as a less restricted estimation method (Li, 2016).

**Table 1:** Statistical estimated parameters for personality CFA.

	**Personality (M1)**			**Personality (M2)**		
	**Estimate (Std.Err.)**	***z***	***p***	**Estimate (Std.Err.)**	***z***	***p***
**Factor loadings**						
**Personality**						
P1	0.06 (0.03)	1.77	0.077	0.06 (0.03)	2.19	0.028
P2	–0.26 (0.04)	–6.25	0.000	–0.26 (0.05)	–5.67	0.000
P3	–0.20 (0.04)	–5.41	0.000	–0.20 (0.04)	–5.57	0.000
P4	0.25 (0.04)	6.23	0.000	0.25 (0.04)	5.67	0.000
**Intercepts**						
P1	1.32 (0.02)	58.67	0.000	1.32 (0.02)	58.60	0.000
P2	1.60 (0.02)	67.56	0.000	1.60 (0.02)	67.48	0.000
P3	1.55 (0.02)	64.54	0.000	1.55 (0.02)	64.47	0.000
P4	1.34 (0.02)	58.66	0.000	1.34 (0.02)	58.59	0.000
**Residual variances**						
P1	0.22 (0.01)	14.42	0.000	0.22 (0.01)	25.03	0.000
P2	0.17 (0.02)	7.91	0.000	0.17 (0.02)	7.15	0.000
P3	0.21 (0.02)	11.62	0.000	0.21 (0.01)	14.57	0.000
P4	0.16 (0.02)	8.08	0.000	0.16 (0.02)	7.23	0.000
**Latent intercepts**						
Personality	0.00^+^			0.00^+^		
**Latent variances**						
Personality	1.00^+^			1.00^+^		
**Fit indices**						
χ^2^(df)	1.88 (2)		0.391	1.42 (2)		0.491
RMSEA	0.00			0.00		
SRMR	0.02			0.02		
CFI	1.00			1.00		
TLI	1.01			1.02		

+ Fixed parameter.

By considering traditional suggestions of cutoff criteria for fit indexes for structural equation models (Hu and Bentler, 1999), the results of Table 1 proved to be satisfactory for both personality model estimations. Our second set of CFA results relate to the leadership practices inventory (see Figure 2B). In Table 2, we proceeded as before. Model 1 shows statistical parameters estimated through the full information maximum likelihood estimation method, and model 2 reports the same parameters estimated through diagonally weighted least squares. Here, the results of the CFA differ depending on the estimation method. Indeed, results from model 1 are far from being conclusive because at least two of its goodness-of-fit indexes suggest that data does not confirm the proposed theoretical structure [i.e., $\chi_{\left(df\right)}^{2}=$ 74.07, *p* = 0.000 and RMSEA = 0.18]. Nonetheless, all goodness-of-fit indexes resulting from model 2 suggest that data confirms the proposed measurement model for leadership.

**Table 2:** Statistical estimated parameters for leadership CFA.

	**Leadership (M1)**			**Leadership (M2)**		
	**Estimate (Std.Err.)**	***z***	***p***	**Estimate (Std.Err.)**	***z***	***p***
**Factor loadings**						
**Leadership**						
L1	1.16 (0.06)	19.70	0.000	1.17 (0.09)	13.30	0.000
L2	1.22 (0.06)	19.93	0.000	1.24 (0.09)	13.36	0.000
L3	0.84 (0.04)	19.09	0.000	0.84 (0.08)	10.95	0.000
L4	1.02 (0.05)	19.47	0.000	1.02 (0.08)	12.35	0.000
L5	1.15 (0.06)	19.82	0.000	1.14 (0.09)	12.61	0.000
**Intercepts**						
L1	7.29 (0.07)	106.53	0.000	7.29 (0.07)	106.41	0.000
L2	7.24 (0.07)	101.13	0.000	7.24 (0.07)	101.01	0.000
L3	8.15 (0.05)	159.52	0.000	8.15 (0.05)	159.33	0.000
L4	7.69 (0.06)	125.14	0.000	7.69 (0.06)	124.99	0.000
L5	7.74 (0.07)	113.65	0.000	7.74 (0.07)	113.52	0.000
**Residual variances**						
L1	0.68 (0.06)	10.95	0.000	0.65 (0.27)	2.41	0.016
L2	0.72 (0.07)	10.80	0.000	0.68 (0.30)	2.23	0.026
L3	0.41 (0.04)	11.67	0.000	0.43 (0.21)	1.99	0.047
L4	0.57 (0.05)	11.65	0.000	0.59 (0.24)	2.46	0.014
L5	0.67 (0.06)	11.11	0.000	0.71 (0.29)	2.45	0.014
**Latent intercepts**						
Leadership	0.00^+^			0.00^+^		
**Latent variances**						
Leadership	1.00^+^			1.00^+^		
**Fit indices**						
χ^2^(df)	74.07 (5)		0.000	2.49 (5)		0.778
RMSEA	0.18			0.00		
SRMR	0.03			0.03		
CFI	0.95			1.00		
TLI	0.90			1.01		

+ Fixed parameter.

### 3.2. Personality-leadership structural analyzes

After confirming the psychometric structure of our two constructs, we now switch the attention to their structural relationship (as depicted in Figure 2C and summarized in Table 3). As expected, Tables 1, 2 show that all factor loadings remained statistically significant under both parametric estimation methods. Nonetheless, the personality-leadership structural relationship proved to be weak either under the FIML estimation method (Est = –0.10, standard error = 0.07, *p* < 0.01) or with the DWLS estimation method (Est = –0.11, standard error = 0.03, *p* < 0.01), as in either case the effect size that the personality has on leadership practices is about just one percent of explained variance (R^2^ = 0.01). By depicting all conceptual dimensions from the leadership practices inventory and all personality dichotomies as nodes of a network, we revealed with red and green arrows all resulting relevant connections (as captured by their positive and negative correlations that proved to be statistically significant) between these two theoretical-related constructs (as illustrated in Figure 2D).

**Table 3:** Statistical estimated parameters for the personality-leadership structural model.

	**Model 1**			**Model 2**		
	**Estimate (SE)**	**R** ^2^	***p***	**Estimate (SE)**	**R** ^2^	***p***
**Factor loadings**						
**Leadership**						
L1	1.15 (0.06)^***^	0.67	0.000	1.18 (0.09)^***^	0.70	0.000
L2	1.21 (0.06)^***^	0.68	0.000	1.25 (0.09)^***^	0.71	0.000
L3	0.84 (0.04)^***^	0.63	0.000	0.83 (0.08)^***^	0.62	0.000
L4	1.02 (0.05)^***^	0.64	0.000	0.96 (0.08)^***^	0.58	0.000
L5	1.15 (0.06)^***^	0.67	0.000	1.14 (0.09)^***^	0.65	0.000
**Personality**						
P1	–0.05 (0.03)	0.01	0.117	–0.03 (0.03)	0.00	0.233
P2	0.26 (0.04)^***^	0.28	0.000	0.26 (0.04)^***^	0.29	0.000
P3	0.19 (0.04)^***^	0.15	0.000	0.18 (0.03)^***^	0.13	0.000
P4	–0.25 (0.04)^***^	0.28	0.000	–0.27 (0.04)^***^	0.32	0.000
**Per-lead**	–0.10 (0.07)	0.01	0.180	–0.11(0.03)^**^	0.01	.001
**Fit indices**						
χ^2^(df)	178.80(26)^***^		0.000	66.73 (26)^***^		0.000
RMSEA	0.12			0.06		
SRMR	0.06			0.06		
CFI	0.90			0.93		
TLI	0.86			0.90		

^+^Fixed parameter.

Statistical significance:

** *p* < 0.01 and

*** *p* < 0.001.

## 4. Discussion

As noted throughout this article, the link between personality and leadership has important implications for organizations and is considered crucial in the management literature (Bass et al., 1990). In this writing, we researched this relationship among hundreds of graduate and undergraduate students in Colombia. We provided fresh evidence of this relationship by assessing the association between the MBTI as a proxy for personality and the LPI as a proxy for leadership-related behaviors. We concluded that the relationship between these two constructs proves to be a weak one according to the standards used in the classic descriptions of summaries of research on psychological theories (Meehl, 1990).

The findings of the present study confirm what other authors have said, that there is a relationship between the different personality traits displayed by individuals and their propensity to manifest or develop leadership related behaviors (Bono and Judge, 2004; D'Alessio, 2008). By combining the four personality dichotomies from the perspective of the psychological types that inspired the MBTI (Gardner and Martinko, 1996) with the five leadership practices from the transformational leadership theory (Posner and Kouzes, 1988), we mapped and evaluated 20 possible theoretical relationships between these two constructs. Only seven out of these 20 potential theoretical relationships proved to be statistically significant, and based on these pairwise relationships, we paved the way to unveil unexplored insights for theory-building purposes.

We noticed that the first personality dichotomy (P1: *Extraversion-Introversion*) only revealed a relevant connection with the second (L2: *inspiring a shared vision*) and fifth leadership practice (L5: *encouraging the heart*). This first relationship means extroverts inspire a shared vision more than introverts do. For the practice of encouraging the heart, extravert types are the ones that tend to motivate their followers more compared to introverts. These findings prove what Colbert et al. (2012) mentioned, that extravert types are more likely than introverts to emerge as leaders. In practical terms, this means that introverts face more difficulties in growing professionally and reaching leadership roles as they need help understanding and dealing whit this issue. A practical implication in this regard might be evident for introverts. They should learn how to express themselves without fearing vulnerable in front of others.

Likewise, the second dichotomy (P2: *Intuition-Sensing*) revealed a relevant connection with the first (L1: *challenge the process*) and second (L2: *inspiring a shared vision*) leadership practices, which means that Intuitives tend to challenge the processes more than sensitives do. For the second practice, intuitive types are the ones that inspire a shared vision more than sensitives. These findings are aligned with the study of Garland and Village (2021), who used the MBTI to classify people in leadership positions and found that intuitive leaders are more visionaries than sensitive type leaders, which relates with Myers and Mccaulley (1985) that mentioned that sensitives have clear guidelines on expected roles and responsibilities. In contrast, intuitive types find opportunities to participate in designing the future; that is, accepting the uncertainty in changing times.

Finally, the fourth personality dichotomy (P4: *Judgment-Perceiving*) proved to be the most relevant personality type as it associates with *encouraging the heart, inspiring a shared vision*, and *challenging the process*. This final relationship means that perceivers are more likely to motivate their followers, describe a possible path for the future, and question the status quo than judgment types do. For example, when facing transitions, perceivers tend to facilitate required changes while judgers might avoid them as they opt for maintaining traditions.

These relevant and significant connections might be the psychological mechanisms psychologists, and other management practitioners use to promote self-awareness of their behaviors and boost team learning and development in organizational settings (Costello, 1993; Penzias, 2020). In other words, these findings have a relevant significance to organizations. Theoretically and practically, any person can be a leader, but they must be aware of their personality to identify their strengths and weaknesses and strategically work on them to improve their leadership practices.

It is also interesting to note those connections that were not relevant, as they have to do with the third personality type (P3: *Thinking-Feeling*) with the third (L3: *enabling others to act*) and fourth leadership dimension (L4: *modeling the way*). The lack of relevant relationships between these conceptual nodes might be fruitful in advancing our knowledge of the different mechanisms by which personality types affect leadership practices, which is promising for teaching leadership skills (Shope et al., 2000). Suppose the way we make decisions (i.e., Thinking vs. Feeling) is an essential element that drives the willingness to become a transformational leader. In that case, this should facilitate leaders to interact with followers as a model that shows commitment to ideals and long-term goals, though this might not necessarily be an instantaneous process. In any case, the set of relationships that we examined in this article deserves other comments. To the best of our knowledge, previous works have focused on literature reviews (Gardner and Martinko, 1996; Brown and Reilly, 2009) and the study of Bess and Harvey (2002) is an exception in terms of describing the statistical behavior of the bimodal empirical distributions that result from using this tool as a data collection technique. In this article, we resumed the conceptual ideas that pinpoint the link between leadership-related behaviors and the MBTI scores that describe personality dichotomies (Waite and McKinney, 2015).

The current research is not free of limitations. Following Meehl's (1990) classic description of summaries of research on psychological theories, the fact of obtaining an effect size of 0.01 between MBTI and LPI should be interpreted as a weak connection that deserves systematic scrutiny from a longitudinal approach; that is, further research should unveil whether the reported effect size here remains statistically stable in other samples that might include university students from Colombia or any other Latin American country. Other limitations refer to the impossibility of assuming any causal relationship between personality and leadership, as the design of this work was not experimental. In this regard, we encourage future researchers to think about possible ways to design more controlled observations to infer causality from these two concepts.

For future studies, we suggest including other variables in the analysis. For example, supervisor experience (i.e., supervising and directing others) might be revealing as it provides another angle to the personality-leadership relationship. We are keenly aware that those with a longer supervisor experience might know how to regulate their behavior when they have to disagree with colleagues and/or persuade peers, supervisors, and collaborators.

## Data availability statement

The datasets presented in the study can be found in online repositories. This data can be found here: https://github.com/jcorrean/MBTI-LEADERSHIP.

## Ethics statement

The studies involving human participants were reviewed and approved by Colegio de Estudios Superiores-Ethical Committee. The patients/participants provided their written informed consent to participate in this study.

## Author contributions

RZ-T conceived the study, collected the data, organized the database, wrote the manuscript, and wrote sections of the manuscript. JC cured the data, analyzed the data, and wrote the results along with the supplemental material. RZ-T and JC revised final version of the manuscript read and approved the submitted version.
